# Are infections in children with juvenile idiopathic arthritis more frequent than in healthy children? A prospective multicenter observational study

**DOI:** 10.3389/fped.2022.917731

**Published:** 2022-08-11

**Authors:** Clara Udaondo, Esmeralda Núñez Cuadros, Sara Murias, Agustin Remesal, Rosa Alcobendas, Concepción Guerrero, Sara Guillen-Martin, Marta Escuredo, Esther Aleo, Daniel Alonso, Alfredo Tagarro, Eloisa De Santiago, Marisol Camacho-Lovillo, Fatima Diaz, Dolores Arenas, Pilar Camacho, Maria Jose Lirola, Mariana Díaz Almirón, Cristina Calvo

**Affiliations:** ^1^Pediatric Rheumatology Unit, University Hospital La Paz, Madrid, Spain; ^2^La Paz Research Institute (IDIPaz), Madrid, Spain; ^3^CIBERINFEC ISCIII, Madrid, Spain; ^4^Pediatric Rheumatology Unit, UCG Pediatría, Hospital Regional Universitario de Málaga, Málaga, Spain; ^5^Pediatrics, CS Tres Cantos, Madrid, Spain; ^6^Pediatrics, Hospital de Getafe, Madrid, Spain; ^7^Pediatrics, CS Parque Europa, Madrid, Spain; ^8^Pediatrics, Fundación para la Investigación del Hospital Clínico San Carlos, Hospital Clinico San Carlos, Madrid, Spain; ^9^Pediatrics, CS Lucero, Madrid, Spain; ^10^Fundación de Investigación Biomédica Hospital 12 de Octubre. Instituto de Investigación 12 de Octubre (imas12), Madrid, Spain; ^11^Department of Pediatrics, Infanta Sofía University Hospital, Madrid, Spain; ^12^Infanta Sofia University Hospital and Henares University Hospital Foundation for Biomedical Research and Innovation (FIIB HUIS HHEN), Madrid, Spain; ^13^Facultad de Medicina, Universidad Europea de Madrid, Madrid, Spain; ^14^Pediatrics, CS Tiro de Pichon, Málaga, Spain; ^15^Pediatric Immunology, Rheumatology and Infectious Diseases Department, Hospital Universitario Virgen del Rocío, Seville, Spain; ^16^Pediatrics, CS San Hilario, Seville, Spain; ^17^Pediatrics, CS Cisneo Alto, Seville, Spain; ^18^Pediatrics, Centro Alcala de Guadaira, Seville, Spain; ^19^Pediatric Rheumatology, Grupo IHP - Instituto Hispalense de Pediatría, Seville, Spain; ^20^Biostatistics, Investigation department, IDIPaz, University Hospital La Paz, Madrid, Spain; ^21^Pediatric Infectious Diseases Department, University Hospital La Paz, Madrid, Spain

**Keywords:** juvenile idiopathic arthritis (JIA), safety, infections, methotrexate, tumor necrosis alpha antagonist, infection rate

## Abstract

**Background:**

Children with juvenile idiopathic arthritis (JIA) might be at a higher risk of infection. Our objectives are to describe and compare infection rates in patients with JIA vs. healthy patients.

**Methods:**

A prospective, multicenter observational study was performed in Spain from January 2017 to June 2019. Patients with JIA from 7 participating hospitals and children without JIA (siblings of patients with JIA, and non-JIA children from primary health centers) were followed up with quarterly questionnaires to record infection episodes. Tuberculosis, herpes zoster, and infections requiring hospital admission were considered severe infections. Rates of infection (episodes/patient/year) were compared using a generalized estimating equations model.

**Results:**

A total of 371 children (181 with and 190 without JIA) were included. The median age was 8.8 years (IQR 5.5–11.3); 75% of the patients with JIA received immunosuppressive treatment (24% methotrexate, 22% biologic, 26% both). A total of 667 infections were recorded; 15 (2.2%) were considered severe. The infection rate was 1.31 (95%CI 1.1–1.5) in JIA and 1.12 (95%CI 0.9–1.3) in non-JIA participants (*p* = 0.19). Age <4 years increased the infection rate by 2.5 times (2.72 vs. 1.12, *p* < 0.001) in both groups. The most frequent infection sites were upper respiratory (62.6% vs. 74.5%) and gastrointestinal (18.8% vs. 11.4%). There were no differences in severe infections (2.5% vs. 2%, *p* = 0.65) between the groups. In children with JIA, younger age and higher disease activity (JADAS71) were associated with a higher infection rate.

**Conclusion:**

We found no differences in the infection rate or infection severity between patients with and without JIA. Most infections were mild. An age younger than 4 years increased the infection risk in both groups. Higher disease activity was associated with a higher infection rate.

## Introduction

Most healthy children have episodes of mild infections during the first years of life. In most cases, these episodes are respiratory or gastrointestinal viral infections. Children with juvenile idiopathic arthritis (JIA) have an allegedly higher risk of infection compared with healthy children because of their underlying condition (1). Treatments used in JIA include corticosteroids, disease-modifying antirheumatic drugs (DMARDs), and biologic agents, all of which can increase the frequency of common mild infections and the risk of severe and opportunistic infections (2).

Soon after the implementation of biological agents in the early 2000s, national and international registries were developed to assess the infectious risk concerning these drugs (3). A recent meta-analysis revealed a slightly increased risk of infections among patients receiving a tumor necrosis factor inhibitor (TNFi) (e.g., etanercept, adalimumab, infliximab, golimumab) compared with patients not exposed to a TNFi (4). Despite growing evidence regarding the efficacy and safety of an increasing number of biological agents (e.g., tocilizumab, anakinra, canakinumab, abatacept) for children with JIA, it is unclear whether these agents increase the risk of infections – or whether the risk is increased to all or to only specific infections. Moreover, there is a proportional relationship between the severity of the disease and the intensity of the treatment administered, and this association might constitute a confounding factor when assessing susceptibility to infections (5).

Therefore, although the available data support the efficacy and safety of biologic agents in controlling inflammatory activity, there remain concerns about the possible increased risk of infections in these patients. Despite the efforts made to date, the literature is still limited and there have been few studies specifically designed to assess the risk of infection in patients with JIA compared with healthy controls (1, 6, 7). At the same time, few studies have measured the frequency of infections in healthy children overall, making it difficult to determine the standards of normality. Thus, the objectives of this study were to prospectively assess infection rates in patients with JIA compared with patients without JIA, and to examine the role of biologic agents, age, treatment, and disease activity in infection risk among patients with JIA.

## Methods

### Study setting and design

Spain’s tax-funded National Health System provides universal health coverage at no cost for the general population. Healthcare delivery is organized in catchment areas, each including a general hospital and several primary care facilities. A prospective, multicenter, observational study was performed in 7 pediatric rheumatology units from various public hospitals and their 7 public referral primary health care centers in several geographical areas of Spain between January 2017 and June 2019. The study procedures were approved by the Ethics Committee of La Paz University Hospital (PI-2609) and all participating centers. All participants’ parents or guardians provided informed consent.

We recruited all individuals aged ≤ 18 years with JIA classified according to International League Against Rheumatism criteria (8) followed at any of the participating pediatric rheumatology units during the study period. We also recruited a comparison group of participants without JIA from 2 convenience groups: siblings of patients with JIA, and children without JIA followed at any of the participating primary care centers. The subgroup of children from the primary care centers were matched 1:1 by age and sex to patients with JIA.

We excluded patients with confirmed or suspected primary immunodeficiencies and children under treatment with more than one biologic agent.

### Study variables and procedures

We recorded information at baseline and every 3 months from all participants. We considered a minimum follow-up time of 9 months to include seasonal variations in infections. The maximum follow-up time was 28 months. Baseline variables recorded included demographics, vaccination status (vaccination against varicella zoster, influenza, and whether the vaccines were up to date or not), date of JIA onset, and current JIA treatment. Physician measures of disease activity in JIA included Juvenile Arthritis Disease Activity Score 71 (JADAS 71) and physician global assessment (visual analog scale or VAS scored from 0 to 10) at baseline, at each follow up visit, and at the end of the study. Follow-up measures included JIA treatment (agent, dose, and changes) and information on infectious episodes. Infectious episodes were recorded by the families in a specific questionnaire designed for this purpose. To record infectious episodes during the follow-up, we designed a specific questionnaire and taught the families how to complete it as part of the baseline procedures. The questionnaire included symptoms, diagnosis, need for medical assistance, antibiotic treatment, need for and duration of hospitalization, and complications. The questionnaire information was retrieved quarterly by a pediatrician at each follow-up appointment (among patients with JIA and their siblings) or via telephone (among the remaining non-JIA participants).

### Definitions

An infectious episode was defined as the presence of fever (> 37.5°C), need for oral antibiotic therapy, or a diagnosis of infection by a physician. Infections were classified for study purposes into the following groups: fever without a source, upper respiratory tract infection accompanied by fever (including acute otitis media, pharyngotonsillitis, laryngitis), lower respiratory tract infection accompanied by fever (bronchitis or pneumonia), pyelonephritis, urinary tract infection, skin and soft tissue infection, acute gastroenteritis, chickenpox/herpes zoster (HZ), herpes simplex, osteoarticular infection, meningitis, tuberculosis, and sepsis. Mild acute conjunctivitis, upper respiratory tract infection without fever (common cold), and episodes of laryngitis or bronchospasm without fever were not considered as infectious episodes for the purpose of this study because of their limited clinical relevance.

Infections requiring hospitalization or intravenous antibiotics/antivirals (5,9–11), opportunistic infections (tuberculosis, HZ, and systemic mycosis (6–12)), and potentially threatening bacterial infections (such as pneumonia, pyelonephritis, or osteoarticular infections) were categorized as severe infections. Of note, all participants were screened for latent tuberculosis infection as well as for chronic hepatitis B and hepatitis C infection before treatment with biological therapy, in accordance with the American College of Rheumatology recommendations (13–15).

The main outcome measures were rates of infection (based on total episodes of infection over the total follow-up time, measured as the number of infectious episodes per patient per year), and the percentage of severe infections among the total infections per group. Data were analyzed to compare infection rates between JIA and non-JIA participants and to evaluate factors possibly related to the risk of infection (age, disease activity, and treatment).

### Statistical analysis

For the description of our sample, discrete variables were summarized as percentages, and continuous variables as means and standard deviation. Median and interquartile ranges were used for describing variables with a non-normal distribution. Participants with and without JIA were compared in terms of age, sex, and vaccinations. Differences in continuous variables between groups were analyzed using Student’s t-test or a non-parametric test, depending on the data distribution. Categorical variables were compared by the chi-squared test or Fisher’s exact test. Finally, we examined the association between JIA and the rate of infection using a multivariable generalized estimating equation (GEE) model, with Poisson distribution and logarithmic link function, given that the same patient could present several episodes. GEE models included variables associated with the exposure and the outcome, as well as variables associated solely with the outcome. P-values were obtained from Type III tests of fixed effects, which contains hypothesis tests for the significance of each of the fixed effects specified in the model. The incidence rate ratio (IRR) per group (with and without JIA) and 95% confidence intervals (95%CIs) were estimated. Two-sided tests were used, and a p-value < 0.05 was considered statistically significant. All statistical analyses were performed using SAS Enterprise Guide 5.1statistical software (SAS Institute Inc., Cary, NC, United States).

## Results

Between January 2017 and June 2018, a total of 371 patients were enrolled, 181 patients with JIA and 190 children without JIA. The non-JIA group consisted of 72 (37.9%) siblings of patients with JIA and 118 (62.1%) children attended in primary care centers. Participants were followed up until June 2019. A total of 29/181 (16%) patients in the JIA group stopped recording the information in the questionnaires before the minimum follow-up time of 9 months and were not included in the final analysis. All the participants in the non-JIA group completed the follow-up for at least 9 months. The final analysis included 152 participants in the JIA group and 190 participants in the non-JIA group. Median follow-up times were 23.9 months (interquartile range [IQR] 17.24–26.27) in the JIA group and 15 months (IQR 11.94–24.19) in the non-JIA group. A total of 667 infectious episodes were recorded, 361 in the JIA group and 306 in the non-JIA group.

The demographic data of the cohort are shown in Table 1. There were more girls in the JIA group (75.1%) than in the non-JIA group (58.9%) (p = 0.001). The median age in both groups was similar (9.2 vs. 8.6), although the number of children younger than 4 years was lower in the JIA group (22/181, 12.2%) compared with the non-JIA group (39/190, 20.5%) (p = 0.035). Regarding immunization status, virtually all patients with JIA (97%) and all children without JIA (100%) had a complete immunization status for their age, according to the Spanish vaccination schedule. Patients with JIA showed a higher influenza vaccine rate at baseline (88.1%) than patients without JIA (73.3%) (*p* = 0.032). However, the chickenpox vaccination rate was lower in the JIA group (70.2%) than in the non-JIA group (83.9%) (*p* = 0.007).

**TABLE 1:** Demographic information of patients included.

	Global (*n* = 371)	JIA (*n* = 181)	Non-JIA (*n* = 190)	*P-value* *
Sex (female), n (%)	248 (66.8%)	136 (75.1%)	112 (58.9%)	0.001
Age (years) Median, IQR	8.8 (5.5 – 11.3)	9.2 (5.8 – 11.7)	8.6 (5.1 – 10.5)	
Age ≤ 4 years	61 (16.4%)	22 (12.2%)	39 (20.5%)	0.035
Minimum follow-up completed	342 (92.1%)	152/180 (84%)	190/190 (100%)	
Follow-up (months) median IQR	19.3	23.9 17.2 – 26.2	15 11.9 – 24.1	
Complete immunization for age	346 (98.9%)	160 (97.6%)	186 (100%)	
Influenzae vaccination at inclusion	22 (83.6%)	89 (88.1%)	33 (73.3%)	0.032
Chickenpox vaccination completed	264 (77.4%)	113 (70.2%)	152 (83.9%)	0.007

JIA = Juvenile idiopathic arthritis. SD = standard deviation, IQR = interquartile range.

*Differences in continuous variables between groups were analyzed using Student’s t-test or a non-parametric test, depending on data distribution. Categorical variables were compared by the chi-squared test or Fisher’s exact test.

The baseline description of the JIA group is detailed in Table 2. Oligoarthritis was the most common JIA subtype (57%), followed by polyarthritis rheumatoid factor negative (23%). Some 75% of patients with JIA were receiving immunosuppressant or immunomodulatory agents; 24% were receiving methotrexate, 22% TNFi, and 25.5% a combination of both methotrexate and TNFi. A total of 13 patients (7.1%) were receiving oral corticosteroids at study enrollment; only 1 patient was receiving over 1 mg/kg/day of prednisone.

**TABLE 2:** Juvenile idiopathic arthritis group description.

	n (%)
**JIA categories** Oligoarthritis Polyarthritis Enthesitis-related arthritis Psoriatic arthritis Systemic arthritis Undifferentiated arthritis	104 (57.5%) 41 (22.7%) 14 (7.7%) 10 (5.5%) 6 (3.3%) 6 (3.3%)
**Treatment at inclusion** None Methotrexate only Etanercept only Methotrexate + etanercept Methotrexate + adalimumab Adalimumab only Other Anakinra Tocilizumab Abatacept Methotrexate + tocilizumab Methotrexate + abatacept	45 (24.9%) 43 (23.8%) 35 (19.3%) 23 (12.7%) 23 (12.7%) 5 (2.8%) 7 (3.9%) 2 2 1 1 1
**Time since diagnosis to inclusion** (months) (median, range)	54 (1 – 171)
**Treatment with corticosteroids** Intra-articular injection in the last 6 months Oral Steroids over 1 mg/kg/day	33 (18.2%) 13 (7.2%) 1 (0.6%)
**History or presence of uveitis at inclusion**	40 (22%)
**Relapse during follow-up** Yes, articular Yes, ocular only	75 (41.4%) 10 (5.5%)
**JADAS71 at baseline** Median, IQR Range	1 (0-3) 0-23

JIA = Juvenile idiopathic arthritis; JADAS71 = Juvenile Arthritis Disease Activity Score 71; IQR = interquartile range.

### Infection rate

**FIGURE 1:**
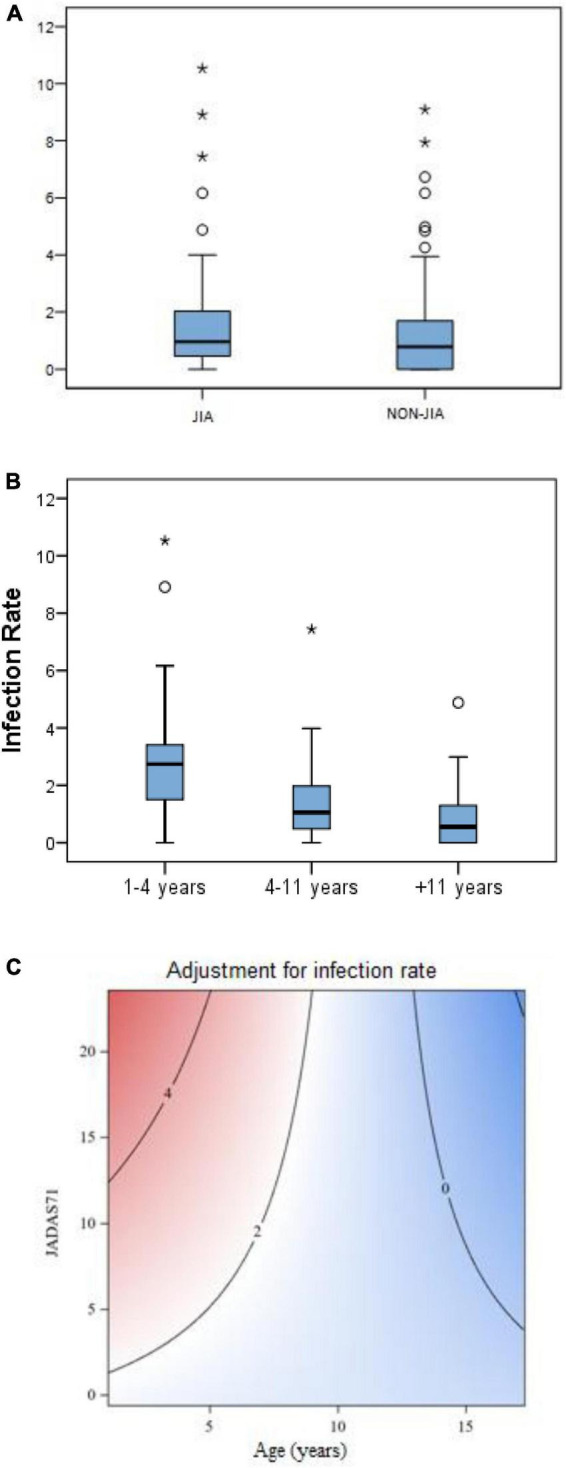
**(A)** Infection rate (episodes/patient/year) in JIA vs. non-JIA. **(B)** Infection rate (episodes/patient/year) according to age bands in both groups. **(C)** Contour plot* - Multivariant analysis graph showing the correlation between infection rate in patients with JIA with age and disease activity (JADAS71). *A contour plot provides a two-dimensional view in which all points that have the same response are connected to produce contour lines of constant responses. A contour plot contains the following elements: **(a)** Predictors for infection rate, displayed on the *x*- (years) and *y*- (disease activity) axes. **(b)** Contour lines that connect points that have the same fitted response value. **(c)** Colored contour bands that represent ranges of the fitted response values (Means for response variables that contain counts that follow the Poisson distribution).

We found no difference in the total infection rate between JIA and non-JIA children (Figure 1A). Patients with JIA had an average rate of 1.31 episodes/year (95% CI 1.1–1.5), while the rate for non-JIA participants was 1.12 (95% CI 0.9–1.3) (Table 3). Globally, children with JIA had 1.16 times more infections than non-JIA children (IRR 1.16, p = 0.19). There were no significant differences in the infection rate between JIA and non-JIA participants after adjusting for sex.

**TABLE 3:** Infectious episodes and rate of infection in both groups.

	Global	JIA	Non-JIA	*P-value* *
Total infections reported (n)	667	361	306	
Infection rate (n° infections/patient/year) (mean, 95% CI)	1.24 1.1 – 1.4	1.31 1.1 – 1.5	1.12 0.9 – 1.3	0.19
Infection rate - age groups Infection rate in ≤ 4 years (mean, 95% CI)		2.72 1.9 – 3.8	2.20 1.7 – 2.9	0.32
Infection rate in > 4 years (mean, 95% CI)		1.12 0.9 – 1.3	0.83 0.7 – 1.0	0.02
**Severe infections** **(n,%)** Hospital admission Gastrointestinal infection Pneumonia Herpes zoster Pneumonia (not hospitalized)	15 (2.2%) 4 (0.6%) 2 2 3 8	9 (2.5%) 3 (0.8%) 2 1 2 4	6 (2%) 1 (0.3%) 0 1 1 4	0.65
Number of episodes/year (n) 0 1-3 4-6 7-10	108 171 44 19	31 86 24 11	77 85 20 8	
**Sensibility analysis**				
Infection rate (JIA vs JIA siblings) (mean, 95% CI)		1.31 1.12 – 1.53	1.0 0.7 – 1.3	0.10
Infection rate (JIA vs. non-JIA excluding siblings) (mean, 95% CI)		1.31 1.1 – 1.5	1.22 0.9 – 1.5	0.60
Infection rate in ≤ 4 years (excluding those > 6episodes/year) (mean, 95% CI)		1.66 1.2 – 2.4	1.45 1.1 – 1.9	0.56
Infection rate in > 4 years (excluding those > 6 episodes/year) (mean, 95% CI)		0.90 0.8 – 1.1	0.77 0.6 – 0.9	0.16
**Effect of age on infection rate in both groups**				

	** ≤ 4 years**	** > 4 years**	*p*-value*	**IRR**
Infection rate (mean, 95% CI) JIA	2.72 1.9 – 3.8	1.12 0.9 – 1.3	< 0.001	2.43
Infection rate (mean, 95% CI) Non-JIA	2.20 1.7 – 2.9	0.83 0.7 – 1.0	< 0.001	2.65

JIA = Juvenile idiopathic arthritis. 95% CI = 95% confidence interval. IRR = Incidence Rate Ratio.

*The analysis was performed using a multivariable generalized estimating equation (GEE) model, with Poisson distribution and logarithmic link function, since the same patient can present several episodes. P-values were obtained from the Type III tests of fixed effects.

Adjusting for age, we found that the infection rate was higher in children aged 4 years or younger, compared with children older than 4 years. This led to a 2.5 fold higher infection rate in younger children compared to children older than 4 years in both groups (IRR 2.4 and 2.6 in JIA and non-JIA, p < 0.001) (Figure 1B and Table 3). Age was the most consistent risk factor for infection, both overall and in each group (JIA and non-JIA participants) separately, and it remained significant across sensitivity analyses (Table 3). Among children aged 4 years or younger, there was no difference in infection rate between JIA and non-JIA participants.

Among children older than 4 years, the infection rate was higher in JIA vs. non-JIA participants (1.12 vs. 0.8), with a 1.4-fold higher risk of infection in JIA compared with non-JIA (IRR 1.4, *p* = 0.02). A sensitivity analysis excluding all cases with 6 or more infectious episodes per year found no age-adjusted differences in the infection rate in JIA vs. non-JIA participants. During the follow-up period, 31 patients with JIA compared with 77 controls had no infectious episodes. Lastly, we conducted a set of additional analyses comparing patients with JIA with siblings and with non-sibling non-JIA children separately. Notably, both comparisons yielded similar results, indicating no between-group differences in infection rates (Table 3).

### Severe infections

We observed 9 and 6 severe infections in the JIA and non-JIA groups, accounting for 2.5% and 2% of infections, respectively (*p* = 0.65). The rates (episodes/patient/year) were not compared due to very low event frequency. Table 3 describes the severe infections. All severe infections resolved completely without sequelae.

### Type of infection and antibiotic therapy

Table 4 shows the specific infection sites in the JIA and non-JIA groups. For both groups, the most frequent were upper respiratory tract infections (62.6% vs. 74.5% in JIA and non-JIA groups, respectively) followed by gastrointestinal infections (18.8% vs. 11.4%). Overall, 36.5% of patients with JIA and 40.8% of children without JIA received antibiotics, with no differences between groups (*p* = 0.23). Upper respiratory tract infections were the main cause of antibiotic prescription (76.2% of all antibiotic prescriptions). However, of all upper respiratory tract infections, only 30% received antibiotics.

**TABLE 4:** Type of infection by study group.

Infection type	Global	JIA	Non-JIA
Upper respiratory tract	454 (68.1%)	226 (62.6%)	228 (74.5%)
Gastrointestinal	103 (15.4%)	68 (18.8%)	35 (11.4%)
Fever without diagnosis	50 (7.5%)	31 (8.6%)	19 (6.2%)
Lower respiratory tract	34 (5.1%)	19 (5.3%)	15 (4.9%)
Skin and soft tissue	13 (1.9%)	7 (1.9%)	6 (2%)
Herpes simplex	5 (0.7%)	4 (1.1%)	1 (0,3%)
Herpes zoster	3 (0.4%)	2 (0.6%)	1 (0.3%)
Urinary tract	5 (0.7%)	4 (1.1%)	

### JIA and rate of infection

In the univariate analyses, age, initial physician global assessment (VASc), and initial JADAS71 score were associated with the infection rate in patients with JIA. We found no differences in infection rate regarding JIA subtype, presence of JIA flares, or treatment with methotrexate, TNFi, or corticosteroids. Infection rate was then adjusted with a multivariate General Linear Model. The predictor variables were age, JADAS71 and the interaction. Other models were analyzed according to the univariate analysis (possible predictors: age, JADAS71, VASc), however, effects were not statistically significant or had a higher value in AIC index.

In the multivariable GEE model, younger age and higher initial JADAS71 score were associated with a higher rate of infection. This effect is represented in the contour plot graph (Figure 1C): The highest infection rates were found in younger children (below 5 years) with higher initial JADAS (over 10). This effect was greater in younger ages and less important in older children, as can be observed in the contour plot graph (Figure 1C).

## Discussion

In this prospective multicenter study with more than 300 children, we observed no significant differences in the rate of infection in a population of patients with JIA compared with a control group followed prospectively over 9 months or more. Importantly, most infections were mild and there were no differences in the rate of severe infections or the type of infections between the groups. Our study suggests that children diagnosed with JIA might not have a significantly higher infection risk than children without JIA. Younger age and higher disease activity were associated with a higher rate of infection in patients with JIA. To the best of our knowledge, this prospective study is the first to compare the risk of infection in patients with and without JIA, including both mild and severe infections.

**The slightly different vaccination rates between patients with and without JIA** could be explained by the disease itself. Once immunosuppressive treatment is started, live virus vaccines (MMR, included in the vaccination schedule) and HZ live vaccination are contraindicated, which explains the lower vaccination rate against HZ in this group, along with the fact that a small percentage of patients with JIA do not have a complete vaccination schedule for their age. Likewise, patients with JIA and those living with them are recommended an annual influenza vaccination, which is not usually performed in the healthy population. This difference could explain the higher proportion of patients vaccinated against influenza in JIA and the relatively high percentage of influenza vaccination in the control group when including JIA siblings (16).

**Mild infections** are common in children, and they can significantly impact quality of life. There is little information in the literature about the rate of mild infections in healthy children, making it difficult to determine the standards of normality. The 2.5-fold higher risk of infection in younger children is highlighted in our study in both JIA and non-JIA. The rate of mild infections in patients with JIA has not been prospectively compared with healthy children as far as we know; most of the existing studies focus solely on patients with JIA and/or severe infections. In a systematic review of infections in patients with JIA treated with TNFi, Toussi et al. found the infection rates in studies ranged from 0.05 to 2.82 episodes per patient per year (2).Consistent with our results, previous studies have found upper respiratory infections to be the most common mild infections in JIA children, affecting 60–72% of patients, followed by gastrointestinal infections in almost 20% (17–19). In children older than 4 years, the infection rate was slightly higher in patients with JIA compared with children without JIA. This difference could be partially explained by the higher number of patients without JIA with no infectious episodes during the follow-up period. It should also be noted that the follow-up period was longer in the patients with JIA. This longer period could be important,because seasonality influences infection rate; however, the analysis could not be adjusted by this fact. Nevertheless, given that the higher rate of infections in patients older than 4 years could not be confirmed across the sensitivity analysis, further studies with larger sample sizes would be needed to confirm this result.

Concerning **severe infections**, in a large retrospective study using national Medicaid data, Beukelman et al. found an increased rate of serious bacterial infections in over 8000 patients with JIA compared withover 300,000 children with attention-deficit/hyperactivity disorder (ADHD) (1). Our results differ from these findings, given that we did not observe any difference in the rate of severe infections in both groups. One explanation could be that, compared with this study, our sample size was small and there were few severe infections, possibly not conferring the statistical power needed to demonstrate significant differences. Nevertheless, in our study,the number of HZ infections was higher in the JIA group (2 vs. 1 in non-JIA participants) and there were more infections requiring hospitalization in patients with JIA (3 vs. 1), suggesting a small but higher risk of serious infection in patients with JIA vs. non-JIA children. Salonen et al. in a retrospective register linkage study comparing the incidence of pneumonia in patients with JIA and healthy controls in Finland found a significantly higher rate of pneumonia from 2007–2014 in patients with JIA compared with age-matched controls (7). In our study, we found the same number of pneumonia cases in the JIA and non-JIA groups, although this number was small.

Regarding **opportunistic infections,** Beukelman et al. found that although they were rare among children with JIA, this group had a higher rate of opportunistic infections than children with ADHD (6). In our cohort, the only opportunistic infection found was HZ, accounting for 1/5 of all severe infections. Similarly, a study from Giancane et al. focusing on the rate of opportunistic infections in a large JIA cohort of over 15,000 patients from the Pharmachild registry found that almost 1/5 of all severe and/or serious infections in patients with JIA on immunosuppressive therapy were opportunistic (12). Consistent with our results, the most frequent opportunistic pathogen in their study was HZ, accounting for 62.2% of all opportunistic infections.

We found a **correlation between disease activity in children with JIA and the rate of infection**, both in the univariate and multivariate analyses. This correlation has been previously reported in adult patients diagnosed with rheumatoid arthritis (20). Regarding children, in line with our results, Thiele et al. found the use of corticosteroids, younger age, and higher JIA-activity as risk factors for infections in a retrospective analysis of over 3000 patients from the German JIA registry (BIKER) (21). In this study, relative risk of infection was higher in patients treated with interleukin-1 and interleukin-6 compared with TNFi. Disease activity undoubtedly influences treatment decisions, so individualizing the risk of infection due to each variable is difficult. In our study, participants with higher JADAS71 at baseline had higher total infection rates. Further analysis of the evolution of disease activity and rate of infection over time could not be performed because of the lack of recorded data in our cohort. Nevertheless, the influence of JIA disease activity upon risk of infection remains unclear and should be studied in larger cohorts.

Previous studies have assessed the **risk of infection in patients with JIA regarding different treatment regimens,** especially TNFi, with contradictory results. In a recent meta-analysis, Aeschliman et al. found no significant increase in serious infections among patients with JIA receiving biologic agents compared with those not receiving biologic agents (22). Similarly, Nagy et al. concluded in another meta-analysis that TNFi therapy slightly but not significantly increased the incidence of infection in JIA children compared with other therapies (4). On the other hand, some prospective studies with large numbers of patients with JIA found a higher risk of bacterial infections in those treated with TNFi compared with DMARDs (17– 23). In our study, we found no differences in infection outcomes in patients with JIA regarding treatment with methotrexate or TNFi. Also, the number of patients with JIA treated with corticosteroids was altogether small in our cohort. This difference might have reduced the risk of infection potentially associated with corticosteroid treatment in patients with JIA. Again, our small sample size could have limited our detection of the influence of treatment upon infection risk, given that this was not the main purpose of the study. Although the population-level risk of infection might be slightly higher according to larger studies, we have not been able to demonstrate this with our sample, suggesting that the risk, if any, is not very high.

One of the **strengths** of this study is that it was a multicenter study performed in Spain, where a tax-funded National Health System provides universal health coverage at no cost, minimizing the risk of selection bias in the JIA sample. Also, the design is the first to prospectively assess infection risk in patients with JIA compared with non-JIA participants. As well as recording the infectious episodes in a specific questionnaire including mild episodes, we recorded all the available data in patient records, which appeared to be a more reliable method for gathering information. Another main strength relies on the design of the study including a long follow-up period (over 12 months in the majority of patients), providing us the opportunity to explore and compare rates of infection in both groups.

Our study has several **limitations.** Siblings of patients with JIA were included to facilitate the recruitment of the control group, although it resulted in the 2 groups being unmatched in sex and age. However, the results have been adjusted by age and sex, and sensitivity analyses have been performed comparing patients with JIA with sibling and non-sibling controls, and no consistent difference in infection rates between JIA and non-JIA could be demonstrated. On the other hand, although we conducted a multicenter study to increase our sample size, it was still small. We did not have sufficient statistical power to detect differences in infection rates in patients with JIA regarding treatment, or to assess differences in severe infections between the groups. Further studies with larger sample sizes are needed to confirm our results and investigate the relationship between risk of infection, disease activity, and treatments for JIA.

In **conclusion**, in this prospective multicenter study of over 300 patients, children with JIA had a similar infection rate to controls without JIA. An age younger than 4 years was independently associated with a 2.5-fold higher infection rate in both groups. Most infections were mild, with no differences in severe infections, types of infection or antibiotic prescription. In children with JIA, younger age and higher initial disease activity (JADAS71) were associated with an increased infection rate. Future studies will help to confirm our findings and further characterize the risk of infection across treatments. In the meantime, this study brings a reassuring message to clinicians and patients and emphasizes the influence of disease activity on infection risk in patients with JIA.

## Data availability statement

The raw data supporting the conclusions of this article will be made available by the authors, without undue reservation.

## Ethics statement

The studies involving human participants were reviewed and approved by Ethical Committee of the Hospital La Paz (PI- 2609). Written informed consent to participate in this study was provided by the participants or their legal guardian/next of kin.

## Author contributions

CC conceived and designed the study. CU, ENC, SM, AR, RA, SG-M, EA, AT, MC-L, and ML recruited and recorded the data of patients with JIA and their siblings. ME, DAl, ES, FD, DAr, and PC recruited and recorded the data of children without JIA. CU had main responsibility for database elaboration and preparation. MDA revised and supervised the database and performed the statistical analysis and elaboration of tables and figures. CU wrote the first draft of the manuscript. CC and ENC were major contributors in revision and final edition of the manuscript. All authors have read and approved the final version of the manuscript.
